# Accurately estimating the length distributions of genomic micro-satellites by tumor purity deconvolution

**DOI:** 10.1186/s12859-020-3349-5

**Published:** 2020-03-11

**Authors:** Yixuan Wang, Xuanping Zhang, Xiao Xiao, Fei-Ran Zhang, Xinxing Yan, Xuan Feng, Zhongmeng Zhao, Yanfang Guan, Jiayin Wang

**Affiliations:** 1 School of Computer Science and Technology, Xi’an Jiaotong University, Xi’an, 710048 People’s Republic of China; 2 Shaanxi Engineering Research Center of Medical and Health Big Data, School of Computer Science and Technology, Xi’an Jiaotong University, Xi’an, 710048 People’s Republic of China; 3 Institute of Health Administration and Policy, School of Public Policy and Administration, Xi’an Jiaotong University, Xi’an, 710048 People’s Republic of China; 4 Department of General Surgery, The First Affiliated Hospital of Shantou University Medical College, Shantou, 515041 Guangdong People’s Republic of China; 5 Geneplus Beijing Institute, Beijing, 100061 People’s Republic of China

**Keywords:** Cancer genomics, Genomic micro-satellite, Length distribution estimation, Tumor purity, Computational pipeline, Sequencing data analysis

## Abstract

**Background:**

Genomic micro-satellites are the genomic regions that consist of short and repetitive DNA motifs. Estimating the length distribution and state of a micro-satellite region is an important computational step in cancer sequencing data pipelines, which is suggested to facilitate the downstream analysis and clinical decision supporting. Although several state-of-the-art approaches have been proposed to identify micro-satellite instability (MSI) events, they are limited in dealing with regions longer than one read length. Moreover, based on our best knowledge, all of these approaches imply a hypothesis that the tumor purity of the sequenced samples is sufficiently high, which is inconsistent with the reality, leading the inferred length distribution to dilute the data signal and introducing the false positive errors.

**Results:**

In this article, we proposed a computational approach, named *ELMSI*, which detected MSI events based on the next generation sequencing technology. *ELMSI* can estimate the specific length distributions and states of micro-satellite regions from a mixed tumor sample paired with a control one. It first estimated the purity of the tumor sample based on the read counts of the filtered SNVs loci. Then, the algorithm identified the length distributions and the states of short micro-satellites by adding the Maximum Likelihood Estimation (MLE) step to the existing algorithm. After that, *ELMSI* continued to infer the length distributions of long micro-satellites by incorporating a simplified Expectation Maximization (EM) algorithm with central limit theorem, and then used statistical tests to output the states of these micro-satellites. Based on our experimental results, *ELMSI* was able to handle micro-satellites with lengths ranging from shorter than one read length to 10kbps.

**Conclusions:**

To verify the reliability of our algorithm, we first compared the ability of classifying the shorter micro-satellites from the mixed samples with the existing algorithm *MSIsensor*. Meanwhile, we varied the number of micro-satellite regions, the read length and the sequencing coverage to separately test the performance of *ELMSI* on estimating the longer ones from the mixed samples. *ELMSI* performed well on mixed samples, and thus *ELMSI* was of great value for improving the recognition effect of micro-satellite regions and supporting clinical decision supporting. The source codes have been uploaded and maintained at https://github.com/YixuanWang1120/ELMSI for academic use only.

## Background

Micro-satellites are repetitive DNA sequences that consist of specific oligonucleotide units [1, 2], exposing intrinsic polymorphisms in terms of the length, which are often described as length distributions [3]. A distinct event known as micro-satellite instability (MSI) refers to a pattern of hypermutation caused by defects in the mismatch repair system [4], characterized by widespread length polymorphisms of micro-satellites repeats, as well as by elevated frequency of single-nucleotide variants (SNVs) [3, 5]. MSI happens if the length distributions of the same micro-satellite region differ significantly between different tissue samples, such as a tumor sample and a normal sample, otherwise the micro-satellite stability (MSS) event exists. Up to 15% – 20% of sporadic cases of colorectal cancer exhibit MSI events [6, 7], while 12% of advanced prostate cancer cases have MSI events [8]. Some recent studies have surveyed the MSI landscape across a range of cancer types [9–11], and imply that these regions have important clinical implications for cancer diagnostics and patient prognosis [12, 13]. For example, MSI positive colorectal tumors respond well to PD-1 blocade [14]. Due to these clinical utility, the detection of MSI events has become increasingly important.

Owing to the increasing prevalence of the next generation sequencing (NGS) technologies, several computational tools for MSI diagnosis utilizing NGS data were developed, replacing the traditional fluorescent multiplexed PCR-based methods, which are time-consuming and costly. These algorithms includes *MSIsensor* [15], *mSINGS* [16], *MANTIS* [17], *MSIseq* [18], *MSIpred* [19], and *MIRMMR* [20]. Based on our best knowledge, these algorithms may be roughly divided into two categories: the read-count distribution based ones and mutation burden based ones. *MSIsensor* is among the first algorithms for analyzing cancer sequencing data, calculating the length distributions of each micro-satellite in paired tumor-normal sequence data and implementing a statistical test to identify significantly altered events between these paired distributions. *mSINGS* works based on target-gene captured sequencing data, allowing for the comparisons among the numbers of signals that reflect the repetitive micro-satellite tracts by differing lengths from tumor and control samples. *mSINGS* is computationally complex, and is thus only suitable for small panels. *MANTIS* analyzes MSI of a normal-tumor sample pair as an aggregate of loci instead of analyzing the differences of individual loci. By pooling the scores of all the loci and focusing on the average score, the impacts that sequencing errors or poorly performing loci may have on the results can be reduced. Meanwhile, *MSIseq*, *MIRMMR* and *MSIpred* utilize machine learning algorithms to predict MSI status. *MSIseq* compares the length distributions using four machine learning frameworks: logistic regression, decision tree, random forest and naive Bayes approach. It is a classifier that only reports MSI-H vs. non-MSI-H, without a score or percentage, or information about the instability of particular loci. *MIRMMR* builds a logistic regression classifier that considers both the methylation and mutation information of the genes belonging to MMR system. *MSIpred* adopts a support vector machine (SVM) to compute 22 features characterizing the tumor mutational load from mutation data in mutation annotation format (MAF) generated from paired tumor-normal exome sequencing data, and then use these features to predict tumor MSI status in the SVM. The classifier was trained by the MAF data of 1074 samples belonging to four types. But none of these approaches is able to overcome the one-read-length limitation. Since the detector can no longer squeeze the micro-satellites by partially mapping reads, the algorithms cannot locally anchor the micro-satellite by using paired-end reads. To this end, *ELMSI* has been proposed to break through this one-read-length limitation.

**Fig. 1:**
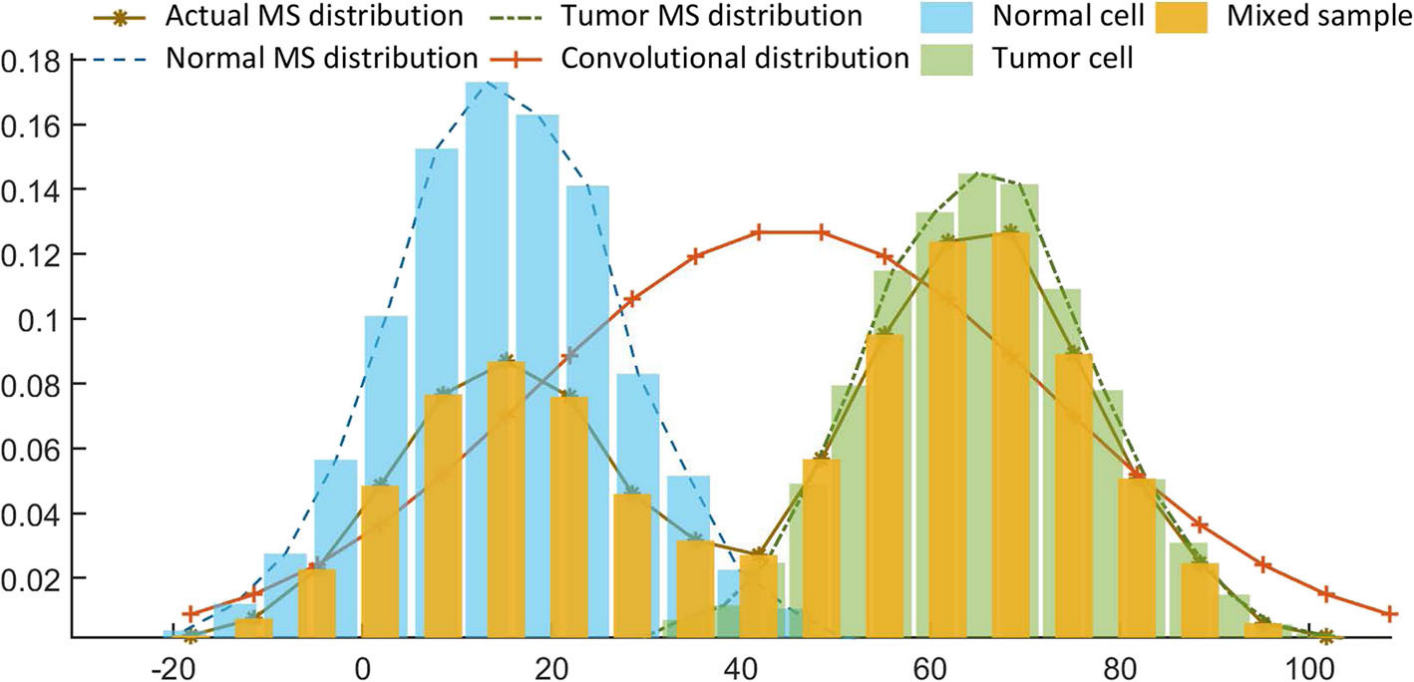
Micro-satellite length distributions in the mixed sample

Of note, all of these existing algorithms generally imply a hypothesis that the tumor purity of the input sequenced samples is sufficiently high, where the purity refers to the proportion of tumor cells in the mixed sample, which varies widely among different samples and cancer types. But in practice, the sample purity is not as high as expected. Due to the growth pattern of tumor tissues and clinical sampling method, the tumor sample sequenced is actually a mixture that contains non-cancerous cells [21]. The presence of non-cancerous cells can influence the judgment of micro-satellite state. Ignoring the tumor purity, the micro-satellite length distributions and states may be inaccurate. For a micro-satellite region from a mixed tumor sample, different tissues may carry different length distributions, while the observed “distribution” from the sequencing data is actually a convolution of the distribution in tumor cells with that in normal cells. If we first established an assumption that the input sample is sufficiently pure, which means we have already assumed that there is only one distribution existing in the mathematical model, then we cannot fit the actual two distributions at all (See Fig. 1). Meanwhile, even if we can use a software to estimate the tumor purity *p* in advance, we cannot directly solve the deconvolution problem. In order to recognize the actual length distribution of the tumor micro-satellite from a given mixed sample, we must calculate the parameter values of the distributions accurately. Furthermore, since the existing algorithms mainly use statistical tests to detect MSI, even if the sample is pure enough, the convolutional distribution inferred based on a set of mixed data containing the normal tissue micro-satellite length data, which will dilute the data signal and may mislead the statistical tests to report a MSS event, introducing type-I error finally. Existing tumor purity estimation algorithms, such as *EMpurity* [22], can accurately identify the proportion of normal cells and tumor cells in sequencing samples respectively, which is helpful for us to further correct the length distributions according to the estimated purity.

Motivated by this, in this article, we proposed a novel algorithm termed *ELMSI* that offers a new approach to identify the state and length distributions of the microsatellite from a given mixed sample. First, we established a more realistic hypothesis that the sequencing sample is a normal-tumor mixed sample, where the micro-satellite lengths are subject to two different distributions. Secondly, we used the purity estimation algorithms to accelerate the deconvolution process for calculating the respective distribution parameters. Finally, our algorithm was suitable for both short and long MSI detection. To test the performance of *ELMSI*, a series of simulation experiments were conducted. Because *mSINGS* is only used for small panels and *MSIseq* targets the sequencing at smaller regions of interest, while *ELMSI* instead focuses on longer micro-satellite and larger panel, these algorithms were not selected for comparisons. The experimental results herein were compared with *MSIsensor*. The results demonstrated that *ELMSI* can accurately identify the state of micro-satellite and infer the length distributions of it from a given mixed normal-tumor sample. Our algorithm outperformed *MSIsensor* based on multiple indicators, maintaining satisfactory accuracy even when coverage decreases at the same time.

## Methods

### Computational pipeline

Suppose that we are given a series of mapped files in BinAry Map (BAM) format generated from a normal-tumor mixed sample, and the outputs of the proposed algorithm include both the length distributions and the state of each micro-satellite. The proposed approach, *ELMSI*, consists of three components. The first component is estimating the tumor purity of the given sequenced sample by calculating the read counts of the filtered SNVs. Based on the estimated purity, the second component identifies the length distributions and the state of the shorter micro-satellites from the mixed sample by adding the Maximum Likelihood Estimation (MLE) step to the existing algorithm *MSIsensor* [15]. The third component infers the length distributions of the longer micro-satellites by combining a simplified Expectation Maximization (EM) algorithm with central limit theorem, and then uses statistical tests to output the states of them. Here, a model of micro-satellite evolution which has been well recognized in recent years holds that the distribution of micro-satellite length is a balance between length mutations and point mutations [23, 24]. Length mutations, the rate of which increases with increasing repeat counts, favor loci to attain arbitrarily high values, whereas point mutations break long repeat arrays into smaller units. Therefore, we make the same assumption [25] that the length distribution approximates a normal distribution. We have made two assumptions on the established computational model:

1. The input sequenced sample is not pure, containing micro-satellites of two types (normal cells and tumor cells) represented by two kinds of length distributions.
2. The length distribution of a micro-satellite approximates a normal distribution.

Before building the model, we need to process the input data. We have the Binary Map format (BAM) files of whole-exome sequencing (WES) data mapped to reference genome by *bwa* [26] as our initial input data. Then, we define the following important terms on the aligned reads.

**MS-pair:** Two paired reads, one of which is perfectly mapped while the other spans a breakpoint.

**SB-read:** A read which is across the breakpoint in an MS-pair.

**PSset:** A collection of the binary group consisting of initial positions and sequences of the SB-reads, which is represented by (POS, SEQ).

**Sk-mer:** The sequence consisting of the first *k* bases.

We first find all the micro-satellite candidate regions by scanning the given reference genome, recording micro-satellites of maximum repeat unit length 6bp and saving the location and the corresponding sequences of each site. Then, we use a clustering algorithm to find the remanent micro-satellite candidate regions which may be ignored by the initial scanning. This algorithm clusters are based on the distances among the initial mapping positions of the reads across each breakpoint. The number of clusters represents the number of micro-satellite regions. We set *L*_*max*_ as the longest length of micro-satellites. The lengths of micro-satellites are generally less than 50kbps [27]. Thus, *L*_*max*_ is set to be 50kbps. *ELMSI* estimates the number of micro-satellites using a clustering algorithm according to the distances of the initial positions of the SB-reads. The clustering strategy is as follows:

According to the mapping results from the PSset, two SB-reads will belong to the same cluster only if the distance between their initial positions is less than *L*_*max*_. Each cluster then represents a candidate micro-satellite region, providing the number of micro-satellites.

Once the number of micro-satellites is determined, for each candidate micro-satellite region, *ELMSI* uses a *k*-mer based algorithm to split each read. As the repeat units that compose micro-satellites are usually less than 6 bps, we set *k*=6 as a default. Starting from the first base of the read sequence, the algorithm detects whether two *k*-mer sequences are identical replicates. This sequence is a candidate repeat unit, and the first base of the sequence is a candidate breakpoint of the micro-satellite. The same operation is conducted for all reads in the micro-satellite region and other candidate areas, taking the mode of the repeat units and breakpoints as the final results.

### Estimating the tumor purity of the sample

First, we introduce a tumor purity estimation algorithm. Due to the limitation of current sequencing technologies, the purity problem is almost inevitable during the actual sampling process, so many algorithms are proposed to solve this problem. Among them, *EMpurity* [22] has established a probability model to accurately estimate the tumor cell proportion in the mixed sample. The observed indicators are the numbers of reads supporting the reference allele and mutation at each site, respectively, while the unknown hidden states include the tumor purity and the joint genotype. *EMpurity* designs a probabilistic model to describe the emission probabilities from the hidden states to the observed indicators and the transition probabilities among the hidden states. This model is solved by an Expectation Maximization algorithm.

*EMpurity* uses the pair-sampled DNA sequencing data as the model input data, and only considers the heterozygous sites with somatic mutations. For one sample in the pair, the set of possible genotype values at each loci is *G*={*AA,AB,BB*}. Let *N,T* and *T*_*M*_ represent the normal sample, virtual pure tumor sample and mixed tumor sample, respectively. Here, the virtual pure tumor sample *T* is actually part of *T*_*M*_. Then, for the paired samples, the set of possible combined genotype values is a Cartesian product, which is *G*×*G*={(*G*_*N*_,*G*_*T*_):*G*_*N*_,*G*_*T*_∈*G*}. For any site *i*, let $ n_{N\_ref}^{i} $ and $ n_{T_{M}\_ref}^{i} $ denote the number of reads supporting the reference allele in the normal sample and mixed tumor sample, respectively, each of which follows a binomial distribution with parameters *μ*_*N*_ and $ \mu _{T_{M}} $. There are only 9 possible joint genotypes, which follow a polynomial distribution with parameter *μ*_*G*_. Considering the bias on read depth, we assume that tumor purity follows a normal distribution across all of the given sites, whose parameters are *μ*_*p*_ and *λ*_*p*_. Let $ R^{i}=\left \{ n_{x\_ref}^{i}, n_{x\_\overline {ref}}^{i} \right \} $ and $ D^{i}=\left \{ n_{x\_d}^{i} \right \}, x \in \{ N, T, T_{M} \} $. Let $ n_{x\_\overline {ref}} $ be the number of reads supporting the mutation in *x*. Let $ n_{x\_d}^{i} $ represent the read depth in *x*. For *x*∈{*N,T*_*M*_}, these values are observed. And then, the estimation of tumor purity is $ \hat {p} = n_{T\_d}^{i} / n_{T_{M}\_d}^{i} $. Let $ \mathcal {G} $ denote the random variable representing the joint genotype $ \left \{G_{(G_{N}, G_{T})}^{i}\right \} $. Let *𝜗* represent the set of unknown parameters, which is $ \vartheta =\left \{ \mu _{N}, \mu _{T}, \mu _{G}, \mu _{p}, \lambda _{p}^{-1} \right \} $. Suppose that $ \mu _{G_{(G_{N}, G_{T})}} $ satisfies $ 0 \leq \mu _{G_{(G_{N}, G_{T})}} \leq 1 $ and $ \sum \nolimits _{G_{N} \in G} \sum \nolimits _{G_{T} \in G} \mu _{G_{(G_{N}, G_{T})}}=~1 $.

This model is solved by an Expectation Maximization algorithm, where the established likelihood function is:

1 $$ \begin{aligned} &L(R, D, \mathcal{G}; \vartheta) =\prod_{i=1}^{I}\prod_{G_{N} \in G}\prod_{G_{T} \in G}\\ &\left[\mu_{G_{(G_{N}, G_{T})}} Bin\left(n_{x\_ref}^{i}|n_{x\_d}^{i}, \mu_{x_{(G_{x})}}\right) N\left(p_{(G_{N}, G_{T})}^{i}|\mu_{p_{(G_{N},G_{T})}^{i}}, \lambda_{p_{(G_{N},G_{T})}^{i}}^{-1}\right)\right]^{G_{(G_{N}, G_{T})}^{i}}\\ &x \in \{N,T\} \end{aligned}  $$

The specific EM iterative process can be referred to *EMpurity* [22].

### Estimating the length distribution parameters of the short micro-satellite

For the shorter (shorter than one-read-length) micro-satellites, the existing algorithms, such as *MSIsensor* [15], can accurately calculate the specific length data and estimate the state of them. However, when the sequenced sample is a normal-tumor mixture, the calculated micro-satellite lengths actually contain both the normal micro-satellite lengths and the tumor micro-satellite lengths, and the state estimated directly is inaccurate. Thus, given a mixed sample with known proportions (normal cells account for (1−*p*), tumor cells account for *p*) and a micro-satellite region belonging to this sample, *MSIsensor* can detect this micro-satellite region, obtaining a set of the lengths *L*={*l*_1_,*l*_2_,...,*l*_*N*_} as a result. *L* is actually a length data set sampled randomly from two samples which are independent of each other and subject to two different normal distribution models. According to the law of large numbers, the data in *L* have a probability of (1−*p*) to be the length of a micro-satellite from normal cells, and the probability of *p* to be that from tumor cells.

Given a micro-satellite region, we assume that its length follows a normal distribution $ N_{1}\left (\mu _{1}, \sigma _{1}^{2} \right) $ when it belongs to normal cells, while the length of it follows a normal distribution $ N_{2}(\mu _{2}, \sigma _{2}^{2}) $ when it belongs to tumor cells. Therefore, the length of this micro-satellite in the mixed sample follows a probability distribution with the density function *f*=(1−*p*)*f*_1_+*pf*_2_, where *f*_1_ and *f*_2_ is the density function of *N*_1_ and *N*_2_ respectively, while *L*={*l*_1_,*l*_2_,...,*l*_*N*_} is the set of lengths obtained from this mixed micro-satellite sample independently. We can get the values of *μ*_1_,*σ*_1_ by separately detecting normal samples (such as blood samples). Under these known conditions, we can use the Maximum Likelihood Estimation (MLE) step to estimate the values of *μ*_2_,*σ*_2_. From the above, the likelihood function is the joint probability density function of the lengths:

2 $$ \begin{aligned} L(\mu_{2},\sigma_{2})&=\prod_{i=1}^{N} f(x_{i}, \mu_{2}, \sigma_{2})\\ &=\prod_{i=1}^{N}\left[(1 - p) \frac{1}{\sqrt{2\pi}\sigma_{1}} exp \left(-\frac{(x_{i} - \mu_{1})^{2}}{2\sigma_{1}^{2}} \right)\right.\\ &\quad +\left. p\frac{1}{\sqrt{2\pi}\sigma_{2}}exp \left(-\frac{(x_{i} - \mu_{2})^{2}} {2\sigma_{2}^{2}} \right)\right] \end{aligned}  $$

The likelihood function actually reflects the probability of generating these length values in *L*. The parameter values in the likelihood function which can maximize this probability are the estimated values we need to calculate:

3 $$ \left\{ \begin{aligned} \frac{\partial L(\mu_{2}, \sigma_{2})}{\partial \mu_{2}} = 0 \\ \frac{\partial L(\mu_{2}, \sigma_{2})}{\partial \sigma_{2}} = 0 \end{aligned} \right.  $$

By this, the estimated values $ \hat {\mu _{2}}, \hat {\sigma _{2}} $ can be obtained. Thus, the length distributions of shorter micro-satellites from a given mixed sample can be recognized, and then we perform a *z*-test to assess the micro-satellite state.

### Estimating the length distribution parameters of the long micro-satellite

**Fig. 2:**
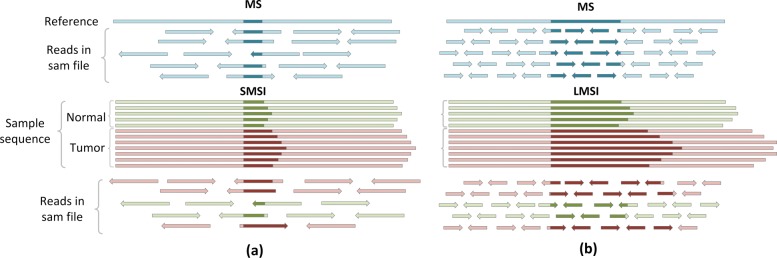
The patterns of sequencing reads from a micro-satellite region sampled from a mixed sample. **a** short MS region. **b** long MS region

On the other hand, for the longer micro-satellites, reads cannot locate them, so we cannot pinpoint their specific lengths. Thus, we use the length distribution to characterize them. Given a mixed sample of normal-tumor cells, we set the proportion of tumor cells as *p* to facilitate the computation. In this paper, we only consider the following two scenarios (See Fig. 2).

**Fig. 3:**
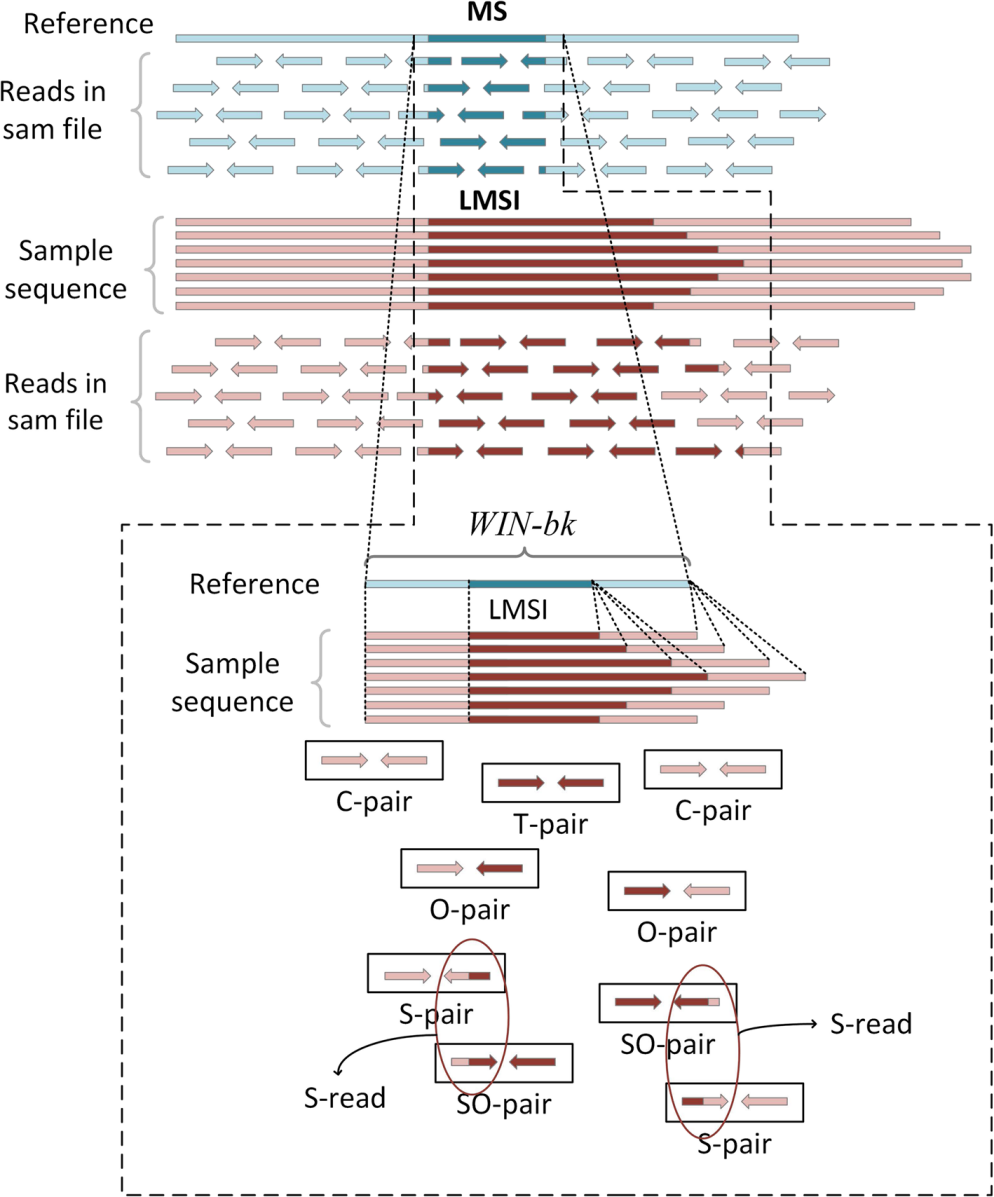
The changes in coverage when a micro-satellite event occurs, and the definitions of different read pairs. C-pairs: The paired-reads in which mapped to WIN-bk; T-pairs: The paired-reads in which both reads mapped from micro-satellite areas; O-pairs: The paired-reads in which one read perfectly matched to WIN-bk and one read mapped from micro-satellites areas; SO-pairs: The paired-reads in which one read is mapped from an micro-satellite area and one read spans across the breakpoints; S-pairs: The paired-reads in which one read is perfectly matched to WIN-bk and one read spans across the breakpoint. S-reads: The reads which span across the breakpoints in SO-pairs and S-pairs

Similarly, we have known that the micro-satellite lengths in (1−*p*) normal cells follow a normal distribution $ N_{1}\left (\mu _{1}, \sigma _{1}^{2}\right) $, while the micro-satellite lengths in *p* pure tumor cells follow an another normal distribution $ N_{2}\left (\mu _{2}, \sigma _{2}^{2}\right) $. And, normal distribution parameters of *N*_1_ can be estimated by detecting normal tissue cells alone. According to central limit theorem, the average of the samples is roughly equal to the average of the population. Whatever the distribution of the population is (mean is *μ*, variation is *σ*^2^), when the sampling times reach a certain condition (>30), the means of the samples (sample size *n*) sampled from it will surround the mean of the population and be normally distributed (mean is *μ*, variation is *σ*^2^/*n*). Due to the specific lengths of longer micro-satellite cannot be assessed by the existing technology, we can use the distribution of the mean length of them to reflect the overall length distribution. Our approach supposes that the length of a micro-satellite is normally distributed. Therefore, *ELMSI* considers a continuous estimation strategy, whose basic goal is to estimate the micro-satellite average length based on the coverage of the specified area containing this micro-satellite, and then using the updated micro-satellite average length to estimate the coverage of this specified area in turn. This loop is repeated until there are no longer significant changes in micro-satellite average length. Therefore, we can use at least 30 groups of sampling average lengths to assess the distribution of the overall long micro-satellite. The length of the hybrid longer micro-satellites belonging to this mixed sample subject to a normal distribution with $ \mu = (1-p) \mu _{1} + p \mu _{2}, \sigma ^{2} = (1-p) \sigma _{1}^{2} + p \sigma _{2}^{2} $. According to the Central Limit Theorem, the sampled average length distribution parameters *μ* can be obtained to reflect the overall length distribution. However, under the technical restrictions, we can only use the estimated *σ*^2^ to represent the overall variance due to the uncountable sample size. By substituting them in the above formula, the length distribution parameters *μ*_2_ and *σ*_2_ of micro-satellites in the pure tumor sample can be calculated. The specific EM process is as follows:

Let *WIN*−*bk* be the window on the reference, with the breakpoint of a micro-satellite as the midpoint of it. The default length of *WIN*−*bk* is set to be 5000bps. Then, the read pairs can be divided into the following categories. Let *C*-pair be the paired-reads perfectly mapped to *WIN*−*bk,T*-pair be the paired-reads perfectly mapped to the micro-satellite region, *O*-pair be the paired-reads with one read mapped to *WIN*−*bk* and the other mapped to the micro-satellite region, *SO*-pair be the paired-reads with one read mapped to the micro-satellite region and the other spanning across a breakpoint, *S*-pair be the paired-reads with one read mapped to *WIN*−*bk* while the other spans across a breakpoint, and *S*-read be the reads which span across the breakpoints in any *SO*-pair or *S*-pair. Figure 3 is a graphical representation of the relevant definitions.

The breakpoints and the repeat units of these micro-satellites can be identified by the aforementioned data preprocessing, we set a *WIN*−*bk* with the breakpoint as the midpoint. The initial length of *WIN*−*kb* is set to be 5000 bps. According to the aligned reads corresponding to *WIN*−*bk*, we can obtain the coverage of reference in *WIN*−*bk* using the following formulas:

4 $$\begin{array}{*{20}l} {SUM}_{{bp}} &= {NUM}_{{read}}\times {L}_{{read}} \end{array} $$

5 $$\begin{array}{*{20}l} {C} &= \frac{SUM_{{bp}}}{L} \end{array} $$

where *SUM*_*bp*_ represents the total number of bases in *WIN*−*bk,NUM*_*read*_ represents the total number of reads in the target area, *L*_*read*_ represents the read length, *C* represents the coverage of the target area, and *L* represents the length of the target area. When the *WIN*−*bk* length is fixed, *SUM*_*bp*_ is a constant. Thus, the lengths of micro-satellites do not affect *SUM*_*bp*_, but do influence the coverage *C*. We can therefore calculate the normal distribution parameters of the micro-satellite lengths through the following nine steps.

1. Variable initialization:
  Let *m* be the total number of micro-satellites, *i* be the *i*th micro-satellites, *S* be the sampling times, *WIN*−*bk* be the sequence of samples with the micro-satellite’s breakpoint as the midpoint, *L*_*Win*_ be the length of *WIN*−*bk,L*_*aln*_ be the total number of bases belong to the micro-satellites region in all *S*-reads, *L*_*set*_ be the set of micro-satellite lengths. Step 1-1: Initializing the number of micro-satellites, the repeating units, breakpoints by the data preprocessing; Step 1-2: Clustering the paired-reads into 5 categories which are: *C*-pairs, *T*-pairs, *O*-pairs, *S*-pairs and *SO*-pairs, all the paired-reads are in *WIN*−*bk*; Step 1-3: Calculating the number of paired-reads in these categories and let *NUM*_*C*_,*NUM*_*T*_,*NUM*_*O*_,*NUM*_*S*_,*NUM*_*SO*_ represent the number of *C*-pairs, *T*-pairs, *O*-pairs, *S*-pairs and *SO*-pairs respectively; Step 1-4: Setting *m* as the number of micro-satellites, *i*=1,*S*=1,*L*_*Win*_=5000*bps,L*^′^=0,*L*_*set*_=*∅*.
2. According to the paired-reads clustering results, calculate the average coverage of *WIN*−*bk*. The formula is $ C = \frac {SUM_{{bp}}}{L}$, where *SUM*_*bp*_=2×(*NUM*_*C*_+*NUM*_*T*_+*NUM*_*O*_+*NUM*_*S*_+*NUM*_*SO*_)×*L*_*read*_+*L*_*aln*_. And *L*=*L*^′^+*L*_*Win*_.
3. Suppose that the coverage follows a uniform distribution, and then the coverage in Step 2 is equal to the coverage in micro-satellite area. In this step, we use the formula $ L^{\prime \prime } = \frac {SUM_{{bp}}}{C}$ to update the micro-satellite length. Where *SUM*_*bp*_=(2×*NUM*_*T*_+*NUM*_*O*_+*NUM*_*SO*_)×*L*_*read*_+*L*_*aln*_.
4. If |*L*−*L*^′′^|>*δ*, where $ \delta = \frac {L^{\prime }}{100} + 1 $, let *L*^′^=*L*^′′^, and repeat Step 2.
5. The obtained micro-satellite length is incorporated into a set, $ L_{{set}} = L_{{set}} \bigcup \{L^{\prime \prime }\} $.
6. In order to assess the normal distribution parameter of a given micro-satellite sequence, we sample 30 times (at least) by changing the size of *L*_*Win*_. Set *S*=*S*+1, if *S*<30, and let *L*_*Win*_=*L*_*Win*_+1000. Then proceed to Step 1.
7. The statistical data regarding micro-satellite lengths obtained from these 30 groups of sampling experiments are tested using a normal test algorithm and the Shapiro-Wilk algorithm. Output the normal distribution parameters of a micro-satellite *N*(*μ*,*σ*^2^). *μ* and *σ*^2^ are the mean and covariance of lengths.
8. If *i*<*m*, set *i*=*i*+1, go to Step 1.
9. The independent *z*-test is used to compare the state of micro-satellite between tumor cells and normal cells. If *p*-value <0.05, then the identified micro-satellite is an MSI event, otherwise the identified micro-satellite is an MSS event.

## Results and discussion

To test the performance of *ELMSI*, we first tested its ability of micro-satellite state classification, and also compared the two major indicators - precision rate and recall rate - with those yielded by *MSIsensor* [15]. And we conducted experiments on a series of simulated datasets with different configurations, which altered the number of micro-satellites, coverage, and read length. In these simulation experiments, the following key indicators were calculated to evaluate *ELMSI*: true positive (TP), false positive (FP), true negative (TN) and false negative (FN). In addition, five popular indicators were further calculated, which are accuracy, recall, precision, MCC and Gain.

1. *Accuracy*=(*TP*+*TN*)/(*TP*+*TN*+*FN*+*FP*);
2. *Recall*=*TP*/(*TP*+*FN*);
3. *Precision*=*TP*/(*TP*+*FP*);
4. $ MCC = (TP \times TN-FP \times FN) / \sqrt {(TP+FP)(TP+FN)(TN+FP)(TN+FN)} $;
5. *Gain*=(*TP*−*FP*)/(*TP*+*FN*).

### Simulation dataset generation

To generate the simulation datasets, we first randomly selected a region of 10Mbps on human chromosome 19. To design a complex situation, we randomly chose the micro-satellites length, repeat unit, and the breakpoint. As aforementioned, the micro-satellite length in a given individual is normal distributed. We divided the normal distribution *N*(*μ*,*σ*^2^) into seven parts which are *μ*−3*σ*,*μ*−2*σ*,*μ*−*σ*,*μ*,*μ*+*σ*,*μ*+2*σ*,*μ*+3*σ*, and the number of micro-satellites in each part planted into the reference was got through multiplied coverage by corresponding probability 1%, 6%, 24%, 38%, 24%, 6%, and 1% for each part, respectively. Once each micro-satellite was planted, we merged these seven read files. All of the simulated reads were then mapped to the reference sequence. The alignment file was then provided to variant calling tools.

### Micro-satellites state classification and comparison experiment

In this part, we first tested the accuracy of *ELMSI* in classifying the micro-satellite state from the mixed samples. The *z*-test was used to determine whether the micro-satellite is a MSI event.

For the shorter micro-satellites, we compared our algorithm with the proposed approach *MSIsensor*. Among the proposed micro-satellite state classification algorithms, *mSINGS* is suitable for small panels and has been reported to be used only for limited exome data, and *MSIseq* only targets the sequencing at smaller regions. Comparison with these algorithms is meaningless. *MSIsensor* can accurately identify the micro-satellite state and lengths when the they are shorter than one read length. Thus we chose *MSIsensor* to do the comparison experiment. The number of micro-satellite was set to be 30, the coverage was set to be 100 × and the read-length was set to be 200bps. The tumor purity was set to be 0.9, 0.7, 0.5, 0.3, 0.1, respectively. Micro-satellite state were subsequently identified by the two classification tools *MSIsensor* and *ELMSI*. The results are shown in Table 1.

**Table 1:** Comparison results of *ELMSI* and *MSIsensor*

Tumor proportion	MSIsensor		ELMSI	
	Precision	Recall	Precision	Recall
0.9	1	0.3333	1	1
0.7	1	0.1333	1	0.6667
0.5	0	0	1	0.5667
0.3	0	0	1	0.6
0.1	0	0	1	0.4667

As can be seen, *ELMSI* has better performance in hybrid micro-satellite state classification. When the tumor purity of the input sequenced sample is below a certain ratio, the MSS signal in the normal sample will dilute the MSI signal, causing *MSIsensor* to report a MSS event. Thus, when the input tumor sample is a mixture with high normal cell contamination, *MSIsensor* cannot distinguish the MSI accurately. However, *ELMSI* can do the classification even if the tumor purity is less than 10%.

On the other hand, for the longer micro-satellites, the paired-reads used to locate the candidate micro-satellite region are invalid, and none of the existing approaches is able to overcome the one-read-length limitation. Thus, we proposed *ELMSI*, which can identify the longer hybrid micro-satellites, and classify their state. Next, we tested the classification accuracy of it. The number of micro-satellite was set to be 30, the coverage was set to be 100 × and the read-length was set to be 200bps. The tumor purity was set to be 0.9, 0.7, 0.5, 0.3, 0.1, respectively. The detailed results are shown in Table 2.

**Table 2:** Performance of ELMSI for longer micro-satellites classification

		Tumor purity			0.9			0.7		
No.	Breakpoint	Unit	*μ*_*N*_	*μ*_*T*_	Breakpoint	*μT*′	MSI	Breakpoint	*μT*′	MSI
1	34489	TCATT	86	125	34491	146.84	1	34489	163.44	1
2	122387	GGCC	425	525	122389	685.63	1	122387	724.23	1
3	189108	GCTAC	46	120	189158	105.65	1	189108	133.03	1
4	190653	CATC	43	136	190655	130.52	1	190653	170.21	1
5	194236	AAC	89	166	194238	145.93	1	194236	151.43	1
6	251655	GCT	71	111	251654	91.17	1	251655	71.71	1
7	311313	ACCA	56	236	311315	321.08	1	311313	331.60	1
8	356789	GCT	76	256	356790	51.47	1	356789	161.35	1
9	398971	TTCG	45	225	398973	213.12	1	398971	251.42	1
10	412340	G	100	280	412505	88.05	1	412340	70.50	1
11	432344	TGA	78	258	432343	177.30	1	432344	220.54	1
12	473174	AAGG	221	354	473176	462.75	1	473174	403.21	1
13	501994	CGCCG	78	161	501996	128.26	1	501994	329.52	1
14	505733	ACAGGG	40	111	505791	222.26	1	505733	248.28	1
15	526358	GTCC	58	144	526360	167.58	1	526358	152.30	1
16	612344	TGC	90	270	612342	355.32	1	612344	343.93	1
17	622735	GGTTC	77	142	622737	114.59	1	622735	197.75	1
18	677621	TCA	70	200	677623	163.89	1	677621	202.36	1
19	712345	GACT	89	269	712337	*N*/*A*	0	712345	230.62	1
20	731506	GA	146	203	731506	104.14	1	731506	88.48	1
21	776166	TAA	213	324	776167	359.1743	1	776166	564.01	1
22	842735	CTC	134	211	842734	213.29	1	842735	236.04	1
23	866450	TG	185	220	866450	371.53	1	866450	526.3551	1
24	891334	TCAGC	105	285	891336	234.20	1	891334	338.38	1
25	908385	AGAAT	167	229	908386	194.85	1	908385	294.27	1
26	910124	C	205	301	910204	98.17	1	910124	32.50	1
27	929056	CCG	120	210	929058	199.72	1	929056	202.66	1
28	944729	GGACT	90	190	944731	214.33	1	944729	225.51	1
29	964608	AGGGGG	56	156	964610	305.59	1	964608	296.61	1
30	973099	GGGCAC	355	460	973101	849.18	1	973099	*N*/*A*	0
					Accuracy		0.967			0.967
Tumor purity		0.5			0.3			0.1		
No.	Unit	Breakpoint	*μT*′	MSI	Breakpoint	*μT*′	MSI	Breakpoint	*μT*′	MS
1	TCATT	34491	155.09	1	34489	165.59	1	34489	272.51	1
2	GGCC	122389	354.08	1	122387	710.49	1	122387	619.04	1
3	GCTAC	189108	177.33	1	189108	127.27	1	189108	94.68	1
4	CATC	190655	137.45	1	190653	84.00	1	190653	19.35	1
5	AAC	194238	172.94	1	194236	125.12	1	194236	82.57	1
6	GCT	251654	86.32	1	251655	30.28	1	251655	*N*/*A*	0
7	ACCA	311355	240.46	1	311313	361.34	1	311313	509.08	1
8	GCT	356790	303.44	1	356789	189.28	1	356789	584.24	1
9	TTCG	398973	275.14	1	398971	282.36	1	398971	295.25	1
10	G	412483	66.43	1	412340	105.09	1	412340	*N*/*A*	0
11	TGA	432343	292.88	1	432344	270.84	1	432344	345.93	1
12	AAGG	473176	434.98	1	473174	640.55	1	473174	779.62	1
13	CGCCG	501996	278.35	1	501994	365.00	1	501994	800.13	1
14	ACAGGG	505844	246.03	1	505733	178.62	1	505733	339.87	1
15	GTCC	526361	120.87	1	526358	132.08	1	526358	112.71	1
16	TGC	612419	467.03	1	612344	570.02	1	612344	*N*/*A*	0
17	GGTTC	622737	215.57	1	622735	241.85	1	622735	254.75	1
18	TCA	677623	226.32	1	677621	241.83	1	677621	220.82	1
19	GACT	712347	393.39	1	712345	327.39	1	712345	*N*/*A*	0
20	GA	731506	77.51	1	731506	51.49	1	731506	*N*/*A*	0
21	TAA	776167	405.81	1	776166	170.12	1	776166	340.20	1
22	CTC	842734	221.52	1	842735	321.37	1	842735	204.91	1
23	TG	866450	485.10	1	866450	870.52	1	866450	236.77	1
24	TCAGC	891336	316.81	1	891334	112.10	1	891334	677.12	1
25	AGAAT	908386	206.39	1	908385	457.04	1	908385	152.63	1
26	C	910220	*N*/*A*	0	910124	*N*/*A*	0	910124	*N*/*A*	0
27	CCG	929058	203.18	1	929056	98.56	1	929056	269.48	1
28	GGACT	944731	257.57	1	944729	250.61	1	944729	279.33	1
29	AGGGGG	964610	416.61	1	964608	380.03	1	964608	691.83	1
30	GGGCAC	973101	1182.00	1	973099	*N*/*A*	0	973099	748.71	1
		Accuracy		0.967			0.933			0.8

As is shown in Table 2, the decreasing tumor ratio can influence the accuracy of the *ELMSI*. However, even with a purity as low as 10%, the results still indicate that *ELMSI* can provide a reliable MSI classification.

### Estimating the distribution of micro-satellite lengths

To separately verify the validity of *ELMSI* in estimating the length distributions of the longer micro-satellites. We ignored the influence of tumor purity, and tested the performance of *ELMSI* by changing micro-satellite number, coverage, and read length. A correct call is defined as follows: a micro-satellites is identified with a correct repeat unit, the breakpoint detected belongs to the (*b*−−10*bps,b*+10*bps*) where *b* is the set breakpoint, and the actual micro-satellites length belongs to the (*μ*−3*σ*,*μ*+3*σ*), where *μ* and *σ* are parameter values which have be estimated.

We first changed the number of micro-satellite from 20 to 100. In order to better reflect the influence of micro-satellite number on *ELMSI*, we also varied the coverage from 30 ×, 60 ×, 100 ×, to 120 ×. The read length was set to be 100bp in this group of experiments. For each different micro-satellite number, we repeated the test five times using the same setting and output the average results, which are summarized in Table 3.

**Table 3:** Key indicators of ELMSI in different numbers number of mciro-satellites

Number of MSIs	Coverage	Accuracy	Recall	Precision	Gain	MCC
20	30 ×	0.5385	0.7000	0.7000	0.4000	-0.300
	60 ×	0.4815	0.6500	0.6500	0.3000	-0.350
	100 ×	0.4419	0.6333	0.5938	0.2000	-0.386
	120 ×	0.5750	0.7667	0.6970	0.4333	-0.265
30	30 ×	0.5250	0.7000	0.6774	0.3667	-0.311
	60 ×	0.5250	0.7000	0.6774	0.3667	-0.311
	100 ×	0.6875	0.8250	0.8049	0.6250	-0.184
	120 ×	0.6400	0.8000	0.7619	0.5500	-0.218
40	30 ×	0.6809	0.8000	0.8205	0.6250	-0.189
	60 ×	0.5882	0.7500	0.7317	0.4750	-0.259
	100 ×	0.5606	0.7400	0.6981	0.4200	-0.280
	120 ×	0.4930	0.7000	0.6250	0.2800	-0.335
50	30 ×	0.6949	0.8200	0.8200	0.6400	-0.180
	60 ×	0.7000	0.8400	0.8077	0.6400	-0.175
	100 ×	0.6000	0.7500	0.7500	0.5000	-0.250
	120 ×	0.5375	0.7167	0.6825	0.3833	-0.299
60	30 ×	0.6164	0.7500	0.7759	0.5333	-0.236
	60 ×	0.6081	0.7500	0.7627	0.5167	-0.243
	100 ×	0.6279	0.7714	0.7714	0.5429	-0.228
	120 ×	0.5862	0.7286	0.7500	0.4857	-0.260
70	30 ×	0.5333	0.6857	0.7059	0.4000	-0.304
	60 ×	0.4787	0.6429	0.6522	0.3000	-0.352
	100 ×	0.5660	0.7500	0.6977	0.4250	-0.274
	120 ×	0.5463	0.7375	0.6782	0.3875	-0.290
80	30 ×	0.6122	0.7500	0.7692	0.5250	-0.240
	60 ×	0.5980	0.7625	0.7349	0.4875	-0.250
	100 ×	0.5664	0.7111	0.7356	0.4556	-0.276
	120 ×	0.5575	0.7000	0.7326	0.4444	-0.283
90	30 ×	0.4957	0.6444	0.6824	0.3444	-0.336
	60 ×	0.5085	0.6667	0.6818	0.3556	-0.325
	100 ×	0.4138	0.6000	0.5714	0.1500	-0.414
	120 ×	0.6667	0.8000	0.8000	0.6000	-0.200
100	30 ×	0.6116	0.7400	0.7789	0.5300	-0.239
	60 ×	0.5191	0.6800	0.6869	0.3700	-0.316
	100 ×	0.6290	0.7800	0.7647	0.5400	-0.227
	120 ×	0.5952	0.7500	0.7426	0.4900	-0.253

The increasing micro-satellite number can influence the robustness of the *ELMSI*. In practice, since microsatellites are very rare, few micro-satellites will exist in a given 10Mbps chromosomal sequence region. Even so, for testing *ELMSI*, we intended to increase this density. Based on Table 3, we can see that *ELMSI* can identify micro-satellites and exclude non micro-satellites interference accurately. The results also show that *ELMSI* can offer a high reliability.

Sequencing coverage affects somatic mutation calling, which in turn would presumably affect the performance of *ELMSI*. To assess the influence of the different coverage on *ELMSI*, we further varied the coverage from 10 × to 100 ×. As is shown in Table 4 the coverage changes intuitively affect the changes in key indicators. In this group of experiments, we set the number of microsatellites to be 20, 40, or 60, and set read length to be 100 bps.

**Table 4:** Comparisons of the performance of ELMSI in different coverages

Number of MSIs	Coverage	Accuracy	Recall	Precision	Gain	MCC
20	10 ×	0.3667	0.5500	0.5238	0.05	-0.4629
	20 ×	0.3846	0.5000	0.5714	0.20	-0.4330
	30 ×	0.4167	0.5263	0.6429	0.26	-0.3974
	40 ×	0.4815	0.6500	0.6500	0.30	-0.3500
	50 ×	0.6957	0.8000	0.8421	0.65	-0.1777
	60 ×	0.6818	0.7895	0.8333	0.63	-0.1873
	70 ×	0.6400	0.7619	0.8000	0.57	-0.2182
	80 ×	0.6071	0.7391	0.7727	0.52	-0.2435
	90 ×	0.5483	0.7083	0.7083	0.42	-0.3077
	100 ×	0.5294	0.6923	0.6923	0.38	-0.3077
40	10 ×	0.3928	0.61110	0.5238	0.06	-0.4303
	20 ×	0.4615	0.6000	0.6667	0.30	-0.3651
	30 ×	0.5106	0.6857	0.6667	0.34	-0.3237
	40 ×	0.5208	0.6756	0.6744	0.38	-0.3148
	50 ×	0.5192	0.6750	0.6923	0.38	-0.3162
	60 ×	0.5762	0.8333	0.7555	0.48	-0.2670
	70 ×	0.6809	0.8000	0.8205	0.63	-0.1895
	80 ×	0.6600	0.8250	0.7674	0.58	-0.2017
	90 ×	0.6538	0.7906	0.7555	0.53	-0.2262
	100 ×	0.6800	0.8500	0.7727	0.60	-0.1846
60	10 ×	0.4218	0.5869	0.6000	0.20	-0.4065
	20 ×	0.4247	0.5167	0.7045	0.30	-0.3779
	30 ×	0.4861	0.6250	0.6862	0.34	-0.3430
	40 ×	0.5441	0.6981	0.7115	0.42	-0.2951
	50 ×	0.5232	0.6818	0.6923	0.38	-0.3129
	60 ×	0.5000	0.6571	0.6765	0.34	-0.3331
	70 ×	0.5455	0.7000	0.7119	0.42	-0.2940
	80 ×	0.5000	0.6470	0.6875	0.35	-0.3321
	90 ×	0.6216	0.7667	0.7667	0.53	-0.2333
	100 ×	0.5444	0.7000	0.7101	0.41	-0.2949

The lower the coverage, the greater the difficulty faced by this computational approach. Consistent with this, Table 4 indicates that the performance of *ELMSI* increases as coverage increases, with maximal recall rate more than 80%. Thus, the higher the coverage, the higher the accuracy of *ELMSI* for inferring micro-satellites.

*ELMSI* can also stay valid when the read length is altered. The number of micro-satellites was set to be 20, or 50, coverage was set to be 30 ×, 60 ×, 100 ×, or 120 ×, and the read length was set to be 100bps, 150bps, 200bps, 250bps and 300bps. The results are shown in Table 5.

**Table 5:** Key indicators of ELMSI corresponding to different read lengths

Number of MSIs	Read length	Coverage	Accuracy	Recall	Precision	Gain	MCC
20	100	30 ×	0.31	0.45	0.5	0	-0.25
		60 ×	0.33	0.5	0.5	0	-0.5
		100 ×	0.31	0.45	0.5	0	-0.52
		120 ×	0.36	0.55	0.52	0.05	-0.46
	150	30 ×	0.31	0.45	0.5	0	-0.25
		60 ×	0.37	0.5	0.59	0.15	-0.45
		100 ×	0.46	0.6	0.6	0.3	-0.36
		120 ×	0.42	0.55	0.65	0.25	-0.40
	200	30 ×	0.5	0.65	0.68	0.35	-0.33
		60 ×	0.49	0.57	0.65	0.1	-0.55
		100 ×	0.48	0.7	0.61	0.25	-0.34
		120 ×	0.52	0.7	0.67	0.35	-0.32
	250	30 ×	0.6	0.75	0.75	0.5	-0.25
		60 ×	0.54	0.7	0.7	0.4	-0.3
		100 ×	0.55	0.75	0.69	0.2	-0.38
		120 ×	0.58	0.75	0.71	0.25	-0.34
50	100	30 ×	0.43	0.6	0.6	0.2	-0.4
		60 ×	0.37	0.55	0.54	0.08	-0.46
		100 ×	0.45	0.63	0.61	0.23	-0.38
		120 ×	0.36	0.55	0.52	0.05	-0.46
	150	30 ×	0.51	0.68	0.63	0.35	-0.33
		60 ×	0.45	0.63	0.61	0.23	-0.38
		100 ×	0.48	0.58	0.62	0.05	-0.45
		120 ×	0.47	0.73	0.63	0.45	-0.28
	200	30 ×	0.51	0.7	0.65	0.45	-0.32
		60 ×	0.61	0.78	0.64	0.5	-0.24
		100 ×	0.52	0.63	0.67	0.15	-0.40
		120 ×	0.56	0.75	0.68	0.4	-0.28
	250	30 ×	0.53	0.7	0.68	0.38	-0.31
		60 ×	0.57	0.78	0.7	0.23	-0.36
		100 ×	0.61	0.7	0.67	0.35	-0.32
		120 ×	0.59	0.75	0.7	0.25	-0.33

The main weakness of this method is the huge amount of splicing required. The longer the read length, the smaller the splicing workload, and the fewer errors will be introduced by splicing. We thus predict that with the increased of read length, *ELMSI* performance will improve. Table 5 validates this hypothesis, and shows that the longer the read length is, the more accurate estimation result is.

## Conclusion

In this article, we focus on the computational problem of inferring the length distributions and states of all kinds of micro-satellites in tumors with normal cell contamination. Existing approaches, such as *MSIsensor*, *mSINGS*, *MANTIS* and *MSIseq*, perform well in handling the genomic micro-satellite event whose length is shorter than one read length, but often encounter a significant loss of accuracy when the length of micro-satellite becomes longer. Meanwhile, all of these MSI detection algorithms implies a general assumption before establishing a mathematical model that the input sample is a pure tumor sample, which is difficult to achieve under existing sequencing technology. We have therefore proposed an algorithm to break these limitations, handling micro-satellites with a wide range of length from a mixed normal-tumor sample based on NGS data. Our proposed algorithm, termed *ELMSI*, directly computes on the aligned reads. *ELMSI* can clearly recognize the length distributions and states of micro-satellites with a wide range of length from mixed sequenced samples. For short microsatellites, it can identify the lengths accurately, while for long micro-satellites, it can estimate the normal distribution parameters. *ELMSI* is among the first approaches to recognize and identify long micro-satellites. However, due to the nature of sequencing data and the limitation of computing capacity, the estimated mean *μ* is relatively accurate, while the estimated variance *σ* has a certain deviation. Thus, for longer MSI detection, our algorithm uses independent *z*-test mainly. When the sample size can be calculated during the iteration process, we can estimate the variance of the longer micro-satellite more accurately, and thus we recommend to use independent t-test to infer the MSI state. The performance of *ELMSI* is compared with *MSIsensor*, and *ELMSI* is superior for the hybrid shorter micro-satellites classification. For the mixed longer samples, *ELMSI* can also obtain the satisfactory results. The simulation experimental results demonstrate that *ELMSI* is robust, with good performance in response to variations in coverage, read length, and the number of micro-satellites. It will be useful for micro-satellites screening and we anticipate a wider usage in cancer clinical sequencing.
